# Alcoholic Mania in Connection with Acute Disease

**Published:** 1881-03-20

**Authors:** 


### ALCOHOLIC MANIA IN CONNECTION WITH ACUTE DISEASE.

[Read by Dr. J. B. Ayer before the Boston Medico-Psychological Society.

According to Dr. Greenfield, the translator of Magnan's work on Alcoholism, there are few medical subjects upon which less has been written than upon acute and chronic alcoholism. The very frequency of the alcoholic element in disease has caused the subject to be neglected.

Verneuil's paper upon the Prognosis of Injuries and Surgical Operations in Drunkards, awakened much interest among the profession. He stated that the deplorable state of the system engendered by alcohol was as important a cause of failure in surgery as exposure to morbid germs. He proved that habitual drinkers who had previously never seemed the worse for liquor, often developed the most serious complications, after receiving insignificant wounds.

He urged that alcoholism, as it so frequently complicates disease, should be studied by the physician no less than by the surgeon.

According to Magnan the habitual drinker ceases to enjoy immunity as soon as the physiological equilibrium is destroyed by the blow of an intercurrent disease.

The following is a summary of a case cited in illustration :

The patient, fifty-one years of age, had, for the past thirty years, stimulated freely without apparent injury to himself, as he always seemed to enjoy the best of health.

The day following more than usual indulgence he was attacked with a catarrhal cold and severe lumbo-abdominal neuralgia. There was nothing unusual about the case until the sixth day, when the fever, pain, and jaundice having disappeared he suddenly developed hallucinations of sight and hearing, and became very suspicious. At first he said little regarding the hallucinations, but as they increased he began to mention them freely, and argued that the pigmies and insects which he saw, and the voices which he heard, were all real, and insinuated that those who did not agree with him were stupid folks.

Muscular tremor, copious perspiration, insomnia and loss of appetite were prominent symptoms.

On the third night after the disappearance of the catarrhal symptoms he was greatly excited, fighting with his imaginary foes. But when he was at the worst he would answer a question correctly, his mind instantly reverting, however, to the old delusions.

On the fourth day, after consultation with Dr.. Jelly, it was decided that full treatment must be carried out. He was accordingly removed to another room. Four doses of chloral and bromide of potash were given at intervals of about an hour up to seventy grains of chloral and eighty-seven grains of bromide, by which time the patient was well under the influence of treatment. Strong beef tea and egg-nogg were insisted upon. This treatment was not carried out without a great deal of urging.

After a two hours' nap the patient awoke with fewer hallucinations. During the following twenty-four hours they gradually lost their hold upon him, and he slept a great portion of the time without further sedative treatment.

He was given ferro-phosphorated elixir of calisaya, and the stimulant was gradually diminished to a little sherry wine and a glass of ale daily with his meals. Although he made a perfect recovery, it was over three weeks before he could give close attention to business without bringing on headache.

While the insomnia, trembling and free perspiration, in connection with the peculiarities of the hallucinations and the possibility of diverting the patient's attention from his delusions pointed strongly to the alcoholic character of the delirium, yet a *positive* diagnosis could not at first be made.

A case was cited, which had been under the reader's care, of a young lady recovering from an attack of peri-uterine cellulitis, who developed hallucinations about bugs, snakes, and Chinese pigmies, and in most respects resembled the previous case. The excitement, however, was not connected with alcoholism. She grew worse and was removed to the South Boston Lunatic Hospital, from which she was discharged well at the end of five months.

The reader advised caution in giving a prognosis in cases of alcoholism, agreeing with Lawson that "the intense furor accompanying alcoholic brain disorder may disappear under treatment in a single night, yet, under precisely the same appreciable conditions, the excitement in another case may continue for weeks, the delirium lasting as long as the deterioration of nutrition and the instability of nerve centres combine to maintain it."

Regarding treatment, bromide of potash and chloral, together with strong beef tea, broths, and (generally) egg-noggs, were advised. Balfour's treatment by forty grains of chloral every hour for three doses (for an adult), combined, if heart is feeble, with one half to one ounce of infusion of digitalis, was commended.

In the discussion which followed, Dr. Webber said that it seemed to him, from the account of the case given by the reader, that the patient was really suffering from delirium tremens, not that it was a case of mania induced by an acute disease in a person addicted to hard drinking. The fact that the patient had been a very hard drinker, and had taken nothing for four or five days, and then was attacked with tremor, sleeplessness, hallucinations of sight and hearing, the objects seen being such as were described, resembling those seen in delirium tremens, renders it probable that that was the disease. The acute disease may, perhaps, have been one element in causing the attack, though the abstinence from alcohol was probably the more influential.

In comparatively mild cases of delirium tremens chloride and bromide of potassium are certainly beneficial. Small doses of these drugs, however, do no good! twenty, thirty, and even forty grains of chloral with thirty to sixty grains of bromide of potassium are more likely to be efficacious in procuring sleep and restoring the patient to sound mind than the smaller doses of ten to fifteen grains, which are sometimes given. The larger doses may be repeated hourly if necessary: One disadvantage of small doses is that the patient seems to be more excited after taking them, and finally a larger quantity of medicine is required. In severe cases, where the pulse is rapid and feeble (120

or more), and where there is great excitement accompanied with hallucinations of sight and hearing, Dr. Webber stated that he had found digitalis the best remedy. He uses the tincture in very severe cases in doses of half an ounce, which may be repeated at the end of four hours if necessary. Many times one dose is sufficient to secure quiet; he has known patients who were greatly excited to fall asleep within half an hour after taking the drug. He would not advise the frequent repetition of these doses. In using digitalis it is all important to observe the pulse; where that is very rapid and feeble in uncomplicated cases large doses can be given; where the pulse is only moderately increased in frequency the larger doses are not called for, one or two drachms being sufficient. Where delirium tremens is complicated with other diseases which have either preceded the mania-a-potu or have occurred at the same time with it, as pneumonia or bronchitis following exposure while the patient was drunk, the treatment must be modified according to circumstances.

Delirium tremens usually occur in old topers or persons who have been addicted to the so-called moderate use, and have at length been on a spree of several days' or weeks' duration. Besides the ingestion of alcohol there is usually also abstinence from food, the patients declaring that they have eaten scarcely any food for one or two weeks, or even longer. There is also usually insomnia, for a longer or a shorter time, and these elements undoubtedly play a part in the delirium tremens.

He said that during the war, in the latter part of 1862, he was stationed on board the receiving-ship Ohio as assistant surgeon; many men were enlisted in the navy who were received on board partially intoxicated, or who had but lately been drinking heavily for a long time; in several instances these men were taken down with delirium tremens after some days of enforced abstinence on board ship. This would show that, contrary to the opinion of some, the sudden breaking off of drinking may be one factor in causing the disease. Most of the patients, however, who are received into the hospital continue to drink up to the time of the attack.

In regard to treatment where there is vomiting, the tincture of capsicum in doses of half a drachm or a drachm is of value, and may be given in combination with the chloral and bromide. After the severity of the atteck has passed off, while the patient is still weak and tremulous, three to five grains of quinine seems to be of use to restore the natural tone of the system.

Dr. George W. Gay then said, this affection is so common in the surgical department of this hospital that we are constantly on the watch for the tremulous tongue and hand, and any undue nervousness in our recent casualty cases. I have also noticed that the wounds of patients threatened with delirium tremens are sensitive out of all proportion to their severity.

Steady drinkers brought here on account of an injury are very apt to show signs of the horrors of delirium tremens, however slight the injury may be. The disease is usually developed in three days, sometimes longer; occasionally it terminates in chronic mania.

The most common treatment here is beef tea well seasoned with red pepper, given as freely as the patient can take it; chloral (fifteen to twenty grains), bromide of potash ammonia (thirty to forty grains), every two or three hours; tincture digitalis in half-ounce doses where the heart is weak. I often preface the treatment with five grains of calomel and fifteen grains bicarbonate of soda. The great majority of cases recover.

Dr. Rowe referred to the well-known fact that patients who had previously received traumatic injuries to the head were peculiarly susceptible to much smaller quantities of alcohol than the ordinary patient. He cited within his knowledge several patients of this class at the Boston Lunatic Hospital who, by very moderate quantities of stimulants, had been made maniacal for varying periods. One case of delusional insanity, having a history of previous head injury, was made excited, sometimes requiring seclusion, by so mild a stimulant as new cider, sometimes served at the social parties given to patients.

Dr. Ray has written a monograph on this feature of brain disease, which can be found in his Contributions to Mental Pathology.

Dr. Rowe, while physician at the House of Correction at South Boston, had tried nearly all reputed remedies in alcoholismus, but found in the majority of cases that nothing served so well as moderately large doses of chloral and bromides. His experience with hyosciamine had been of no avail, but deemed the particular samples used by him as not true hyosciamine.

Dr. Bancroft: In reference to the ætiology of an attack of delirium tremens, two cases occurred under my observation at the City Hospital which seemed to show that the attack did not occur immediately after a prolonged spree, nor after the withdrawal of the daily quantum of liquor; but upon the indulgence in a single glass of whisky after a total abstinence of four or five days. The patients were doing well, but after partaking of four or five ounces of liquor surreptitiously introduced by their relatives, they immediately developed violent attacks of delirium tremens, of which one of them died.

Dr. Bancroft described the case of a young man aged twenty-five, who has recently died at the New Hampshire Insane Asylum. The facts, in brief, are these: in the year 1877 he suddenly shot a man, without having given much evidence of approaching mental bisturbance at that time. Was committed to jail, and during his first day's residence there broke out into a wild mania. His insanity being unquestionable, he was at once sent to the asylum. From the date of his commital, May 24, 1877, until October 12, 1880, he did not utter a single intelligible, connected sentence. He was in almost constant motion, tearing his clothes, filthy in the extreme, and requiring the continuous attention of the attendant. The incoherency of his speech was simply indescribable; one thought had scarcely any connection with another.

For three years he remained in this state. On the 12th of October, after a period of unusually wild excitement, the attendant entered his room and bade him "Good morning;" the patient turned about suddenly and said: "I want to die. I feel ready to die. I have suffered for three long years, and I want to go where I can rest." These were literally the first intelligent words the patient had uttered for three years. He repeated these words a number of times during the next few hours. But he seemed gradually sinking. Occasionally he rallied, and there seemed to be something on his mind that he wished to say. He did speak, among other things, of his wife and of trouble he had had with her, and his daughter, giving the name and age of the latter.

When the attendant went out of the patient's room the latter would call him back, and say there was something he wanted to tell him, but he did not seem able to express himself.

He gradually failed, and died the following day.—*Boston Medical and Surgical Journal.*

